# Anhydrous Ammonia Chemical Release — Lake County, Illinois, April 2019

**DOI:** 10.15585/mmwr.mm6904a4

**Published:** 2020-01-31

**Authors:** Jared R. Rispens, Sydney A. Jones, Nakia S. Clemmons, Sana Ahmed, Laurel Harduar-Morano, Mark D. Johnson, Charles Edge, Aditi Vyas, Ellie Bourgikos, Maureen F. Orr

**Affiliations:** ^1^Epidemic Intelligence Service, CDC; ^2^Division of Environmental Health Science and Practice, National Center for Environmental Health, CDC; ^3^Connecticut Department of Public Health; ^4^Division of Toxicology and Human Health Sciences, Agency for Toxic Substance and Disease Registry, Atlanta, Georgia; ^5^Communicable Disease Prevention, Lake County Health Department and Community Health Center, Waukegan, Illinois; ^6^Division of Surveillance, Hazard Evaluations, and Field Studies, National Institute for Occupational Safety and Health, CDC; ^7^Division of Community Health Investigations, Agency for Toxic Substances and Disease Registry, Atlanta, Georgia; ^8^Department of Occupational and Environmental Medicine, University of Illinois-Chicago.

On April 25, 2019, a farm tractor towing two 2-ton ammonia tanks on a county road in Lake County, Illinois, experienced a mechanical failure that resulted in the release of anhydrous ammonia, a colorless, pungent, irritating gas that can cause severe respiratory and ocular damage (*1*). Approximately 80% of anhydrous ammonia produced in the United States is used as a fertilizer in agriculture (*1*). Eighty-three persons, including first responders, motorists, and neighborhood residents, were evaluated at area hospitals because of exposure to the gas. Two weeks after the release, the Agency for Toxic Substances and Disease Registry (ATSDR) and CDC’s National Center for Environmental Health (NCEH) collaborated with the Lake County Health Department and the Illinois Department of Public Health on an investigation using ATSDR’s Assessment of Chemical Exposures program to describe the release, review the emergency response, and determine health effects associated with the exposure. First responders, community residents, and hospital personnel reported communication challenges related to the nature of the gas release and effective protective measures. Among the 83 persons evaluated at six area hospitals for effects of the chemical release, 14 (17%) were hospitalized, including eight (10%) who were admitted to the intensive care unit (ICU), seven (8%) of whom required endotracheal intubation and mechanical ventilation; no deaths occurred. In addition, ICU health care providers experienced symptoms of secondary exposure. The National Institute for Occupational Safety and Health’s Emergency Responder Health Monitoring and Surveillance Program has specific recommendations and tools to protect responders during all phases of a response (*2*). Hospitals also need to review institutional policies and procedures for chemical mass casualty events, including decontamination (*3*). Prompt and correct identification of hazardous material (hazmat) events, and clear communication among responding entities, including on-scene and hospital responders, is important to ensure effective response after a chemical release.

At 4:24 a.m. on April 25, 2019, a farm tractor experienced a mechanical failure that involved its two ammonia tanks while on a main two-lane county road, resulting in the release of at least 500 gallons (at least 1,893 L) of anhydrous ammonia (each tank had a capacity of 1,000 gallons [3,785 L], but neither was full at the time of the incident). Release of the ammonia created a large, low-lying plume of white gas, which, because of cool, humid air and calm winds, lingered in the area and surrounded nearby homes. Vehicles encountering the plume stalled (possibly caused by the effects on engines or electronics), and drivers and passengers were overcome by the gas, reporting an acrid smell and taste, throat irritation, coughing, difficulty breathing, and choking (Supplementary Figure, https://stacks.cdc.gov/view/cdc/84423). Overall, 129 fire personnel from 39 departments, 30 law enforcement officers from eight departments, and numerous dispatchers and 9-1-1 operators from multiple centers responded. Victims were rescued from cars and homes nearest to the release. A shelter-in-place order (to remain indoors, close all doors and windows, and shut off home heating, ventilation, and air conditioning systems) was issued to residents living within a 1-mile radius of the release and was transmitted by reverse 9-1-1, a system used to notify residents in emergency situations. The fire department applied a water spray to dilute the plume until the ammonia tanks were empty, which occurred at 7:23 a.m. The local fire department, the National Transportation Safety Board, and the U.S. Environmental Protection Agency investigated the release. The National Transportation Safety Board has released a preliminary report of their investigation (*4*).

Ten days after the release, on May 9, 2019, a team from ATSDR and CDC arrived in Illinois to assist the Lake County Health Department and the Illinois Department of Public Health with the Assessment of Chemical Exposures investigation, which uses a toolkit of modifiable surveys to conduct rapid assessments after large-scale toxic substance releases (*5*). This investigation included the following five components: 1) environmental evaluation of the size of the release; 2) abstraction of medical records to characterize the health effects of the release; 3) a survey of first responders who were in or near the plume; 4) a household survey of persons who lived in the four census blocks adjacent to the release; and 5) a survey of hospital emergency department (ED) personnel who treated patients.

## Environmental Survey

To characterize the location and size of the chemical release, the locations of coniferous trees visibly damaged by the ammonia release were mapped as a proxy for the location of the anhydrous ammonia plume. Eighty-one damaged coniferous trees were identified in the release zone, including 59 (72%) with >10 ft (>3 m) of vertical damage. These data suggested that the ammonia plume likely covered at least 0.053 mi^2^ (at least 0.137 km^2^ [1,486,447 ft^2^ (138,095 m^2^)] and extended up to 15 feet (up to 4.5 m) above the ground, especially in areas closer to the release site (Figure). Of note, the plume extended into the nearby golf course but could not be mapped because of the lack of coniferous trees on the course.

**FIGURE:**
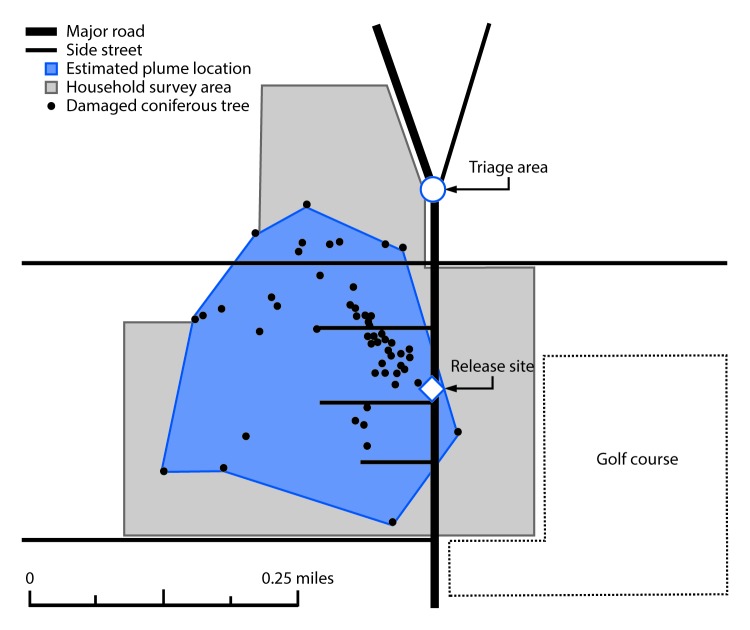
Anhydrous ammonia release site, estimated plume location based on damaged coniferous trees,* and area covered by the household survey for the assessment of chemical exposures — Lake County, Illinois, April 2019 * The plume extended into the nearby golf course but could not be mapped because of the lack of coniferous trees on the course.

## Medical Record Abstraction

At the time of the CDC/ATSDR investigation, medical charts were reviewed for 83 patients evaluated at EDs with complaints related to anhydrous ammonia exposure within 24 hours after the incident (Table 1). Forty-four (53%) patients were female; median age was 34 years (range = 1–79 years). Among 35 persons transported to the ED by emergency medical services (EMS), 15 (42%) were civilians exposed at home, seven (19%) were civilians exposed in their car, and 13 (39%) were first responders. Fourteen patients (including one first responder) were admitted to hospitals, including eight admitted to an ICU, seven of whom were intubated and required mechanical ventilation. Among the eight admitted to an ICU, the duration of their stay ranged from 1 to 7 days (total = 27 ICU days). No deaths occurred.

**TABLE 1:** Characteristics of 83 patients exposed to anhydrous ammonia and treated at six hospitals within 24 hours — Lake County, Illinois, April 2019*

**Characteristic**	**No. (%)**
**Exposure location**	
Home	29 (35)^†^
Car	25 (30)^§^
Work	14 (17)^¶^
Unknown/Missing	15 (18)**
**Hospital admissions**	
Evaluated in ED and discharged	69 (83)^††^
Admitted to general medicine/observation	6 (7)
Admitted to ICU	8 (10)^§§^
Intubated/Mechanically ventilated in ICU	7 (8)^§§^
**Sex**	
Male	39 (47)
Female	44 (53)
**Age (yrs), median (range)**	**34 (1–79)**
**Underlying condition**	
Asthma	10 (12)
Hypertension	9 (11)
Tobacco use	10 (12)
Pregnant^¶¶^	3 (7)
Symptoms***	
Shortness of breath	35 (42)
Cough	27 (33)
Upper airway pain	22 (27)
Headache	15 (18)
Dizziness	13 (16)
Chest tightness	10 (12)
Chest pain	8 (10)
Eye pain	8 (10)
Wheezing	7 (8)
Nausea	7 (8)
Pleuritic chest pain	5 (6)

**Abbreviations:** ED = emergency department; EMS = emergency medical services; ICU = intensive care unit.

* Based on medical chart abstraction data.

^†^ Includes 15 patients (52%) transported by EMS.

^§^ Includes seven patients (28%) transported by EMS.

^¶^ Includes 12 patients (86%) transported by EMS.

** Includes one patient (7%) transported by EMS.

^††^ Includes 12 patients (17%) who were first responders.

^§§^ Includes one first responder.

^¶¶^ Percentage calculated among women.

*** Symptoms are not mutually exclusive. Patients might have had more than one symptom.

## First Responder Survey

Thirty-eight first responders suspected of entering the plume were surveyed (Table 2); 18 (47%) reported entering the plume, five (13%) reported having been near the plume, 11 (29%) did not enter the plume, and the exposure status of four (11%) was unknown. Because dispatchers initially reported the incident as a car fire, some first responders arriving at the scene who were unaware of the chemical release were also overcome by the gas. Other responders who smelled ammonia and saw the white plume retreated several blocks to don a self-contained breathing apparatus before attempting rescues. Among the 18 first responders who entered the plume, nine experienced symptoms of illness within 24 hours of the release; the four most common symptoms, each reported by five responders, were cough, burning lungs, shortness of breath, and eye irritation. Thirteen first responders were transported by EMS to the hospital; many of these responders were evaluated as a precautionary measure. One first responder was hospitalized, requiring intubation, mechanical ventilation, and ICU care.

**TABLE 2:** Exposure location, hospital care, and symptoms experienced by firefighters, police officers, and hazardous materials (hazmat) first responders who entered the gas plume after anhydrous ammonia release and residents from 23 homes near the release site — First Responder and Household Surveys, Lake County, Illinois, April 2019

Characteristic	No. (%)
**First responder survey (N = 38)**	
**First responder type**	
Firefighter/EMS	22 (58)
Police officer	4 (11)
Hazmat	12 (32)
**Exposure location**	
Entered plume	18 (47)
Near plume	5 (13)
Did not enter plume	11 (29)
Unknown/Missing	4 (11)
Reported symptoms* after entering plume (n = 18)	
Yes	9 (50)
No	9 (50)
**Hospital care (n = 13)**	
Evaluated in ED and discharged	12 (32)
Admitted to general medicine/observation	0
Admitted to ICU	1 (2.6)
Intubated/Mechanically ventilated	1 (2.6)
**Household survey (N = 48)**	
**Age group (yrs)**	
Median (range)	53 (1–84)
1–18	5 (10)
>18	43 (90)
**Sex**	
Male	16 (33)
Female	32 (67)
**Hispanic ethnicity**	17 (35)
**Response to shelter-in-place order**	
Remained at home until order lifted	33 (69)
Evacuated by EMS or private vehicle	11 (23)
Left before order lifted	4 (8)
**Symptom^†^/Treatment**	
Experienced symptoms within 24 hours	21 (44)
Cough	15 (71)^§^
Burning nose/throat	14 (67)^§^
Shortness of breath	12 (57)^§^
Eye irritation	12 (57)^§^
Received medical care	15 (31)^§^
ICU admission, intubation/mechanical ventilation	2 (4)

**Abbreviations:** ED = emergency department; EMS = emergency medical services; ICU = intensive care unit.

* Symptoms included cough, burning lungs, shortness of breath, and eye irritation.

^†^ Symptoms are not mutually exclusive. Patients might have had more than one symptom.

^§^ Percentage of those with symptoms.

## Household Survey

Forty-eight residents from 23 homes near the release site were surveyed; their median age was 53 years (range = 1–84 years), and 43 (90%) were aged >18 years (Table 2). Thirty-two (67%) surveyed residents were women, and 17 (35%) were Hispanic. Adult respondents reported initially receiving information about the release from first responders, relatives, friends, neighbors, and reverse 9-1-1. On the morning of the release, 33 (69%) residents remained at home until the shelter-in-place order was lifted, 11 (23%) evacuated from their homes by EMS or in their private vehicles, and four (8%) left home before the order was lifted to go to work or for other reasons. Twenty-one (44%) surveyed residents experienced symptoms of illness within 24 hours after the release; the four most common symptoms were cough (15), burning in the nose or throat (14), shortness of breath (12), and eye irritation (12). Fifteen (31%) surveyed residents reported having received medical care from hospitals, EMS, or other providers, including eight who had evacuated from their homes, two of whom required intubation, mechanical ventilation, and ICU care.

## Hospital Survey

ED administrators at the six hospitals that received patients from EMS during the incident (range = 1–33 patients per hospital) were surveyed. Three hospitals activated internal disaster response measures during the incident. Five reported receiving insufficient information from the scene, especially regarding the type of chemical exposure and the number of patients and their conditions. Because of limited information about the nature of the anhydrous ammonia exposure, initial calls by hospitals to the poison control center resulted in inadequate and incomplete recommendations, especially regarding decontamination. No patients had been decontaminated in the field. Three hospitals decontaminated patients at the hospital (clothing removal and soap/water shower). Two hospitals decontaminated patients upon arrival to the ED, and one hospital began to decontaminate admitted patients after five ICU staff members experienced symptoms of secondary exposure in the ICU from off-gassing* of anhydrous ammonia from victims’ clothing.

## Discussion

Clear communication during a chemical release is essential to reduce exposures and harm. The timing of this event in the early morning when traffic was sparse and its location in a less populated area minimized morbidity among residents and motorists, and the actions of the first responders likely saved lives. However, multiple communication challenges during each part of the response hindered effective action to prevent exposures. Responders who initially arrived on scene were unaware it was a hazmat incident. Although some first responders did don the recommended personal protective equipment after smelling ammonia, half of those who entered the plume experienced symptoms, including one responder who required mechanical ventilation. Most hospitals reported receiving insufficient information about the chemical, type of exposure, and the number and triage category of inbound patients. After relaying this incomplete information to the poison control center, hospitals received inadequate decontamination recommendations, leading to secondary exposures of hospital personnel. After sharing findings from the Assessment of Chemical Exposures investigation, the poison control center reviewed communication flow protocols and reexamined ammonia guidelines to improve decontamination recommendations.

To improve future responses and reduce communication challenges, the assessment team recommended standardizing the 9-1-1 operator training for hazmat events; consolidating 9-1-1 systems or adopting shared computer-assisted dispatch systems; and implementing a comprehensive hazmat communication model to include multi-agency training that incorporated communication with hospitals. Although the probability of hazmat incidents of this magnitude is low, the incidents are of high consequence and threaten first responders and the public. Multi-agency hazmat trainings will improve communications, identify communication gaps, and clarify each agency and responder’s role during a response. The National Institute for Occupational Safety and Health’s Emergency Responder Health Monitoring and Surveillance Program has specific recommendations and tools to protect responders during all phases of a response (*2*). Hospitals also need to review institutional policies and procedures for chemical mass casualty events, including decontamination (*3*). Prompt and correct identification of hazmat events, and clear communication among responding entities, including on-scene and hospital responders, is important to ensure effective response after a chemical release.

**Summary**

**What is already known about this topic?**

Exposure to anhydrous ammonia gas can cause severe respiratory and ocular damage.

**What is added by this report?**

At least 500 gallons (1,893 L) of anhydrous ammonia gas was released from two tanks towed by a farm tractor in a residential area, resulting in evaluation of 83 persons at local emergency departments. Fourteen persons were hospitalized, including seven patients with respiratory failure.

**What are the implications for public health practice?**

Preparation for hazardous materials (hazmat) responses should ensure 1) timely and informative public communication, 2) effective communication among first responders, 3) accurate field information provided to health support personnel, and 4) regular hazmat exercises for all response and support personnel.

## Footnotes

* The evaporation of volatile organic compounds into the air from a contaminated source such as clothing.
